# Effectiveness of Fecal Microbiota Transplantation for Weight Loss in Patients With Obesity Undergoing Bariatric Surgery: A Randomized Clinical Trial

**DOI:** 10.1001/jamanetworkopen.2022.47226

**Published:** 2022-12-16

**Authors:** Perttu Lahtinen, Anne Juuti, Markku Luostarinen, Leo Niskanen, Tarja Liukkonen, Jyrki Tillonen, Jyrki Kössi, Vesa Ilvesmäki, Mikko Viljakka, Reetta Satokari, Perttu Arkkila

**Affiliations:** 1 Department of Gastroenterology, Päijät-Häme Central Hospital, Lahti, Finland; 2 Department of Gastrointestinal Surgery, Helsinki University Central Hospital, Helsinki, Finland; 3 Department of Gastrointestinal Surgery, Päijät-Häme Central Hospital, Lahti, Finland; 4 Department of Endocrinology, Päijät-Häme Central Hospital, Lahti, Finland; 5 Department of Nutrition, Päijät-Häme Central Hospital, Lahti, Finland; 6 Department of Endocrinology, Päijät-Häme Central Hospital, Lahti, Finland; 7 Human Microbiome Research Program, Faculty of Medicine, University of Helsinki, Helsinki, Finland; 8 Department of Gastroenterology, Helsinki University Hospital, Helsinki, Finland

## Abstract

**Importance:**

Severe obesity is a major health concern. However, a few patients remain resistant to bariatric surgery and other treatments. Animal studies suggest that weight may be altered by fecal microbiota transplantation (FMT) from a lean donor.

**Objective:**

To determine whether FMT from a lean donor reduces body weight and further improves the results of bariatric surgery.

**Design, Setting, and Participants:**

This double-blinded, placebo-controlled, multicenter, randomized clinical trial was conducted in 2018 to 2021 among adult individuals with severe obesity treated at 2 bariatric surgery centers in Finland and included 18 months of follow-up. Patients eligible for bariatric surgery were recruited for the study. Data were analyzed from March 2021 to May 2022.

**Interventions:**

FMT from a lean donor or from the patient (autologous placebo) was administered by gastroscopy into the duodenum. Bariatric surgery was performed 6 months after the baseline intervention using laparoscopic Roux-en-Y gastric bypass (LRYGB) or laparoscopic sleeve gastrectomy (LSG)

**Main Outcomes and Measures:**

The main outcome was weight reduction measured as the percentage of total weight loss (TWL).

**Results:**

Forty-one patients were recruited to participate in the study and were included in the final analysis (29 women [71.1%]; mean [SD] age, 48.7 [8.7] years; mean [SD] body mass index, 42.5 [6.0]). A total of 21 patients received FMT from a lean donor, and 20 received an autologous placebo. Six months after FMT, 34 patients underwent LRYGB and 4 underwent LSG. Thirty-four patients (82.9%) attended the last visit 18 months after the baseline visit. The percentage of TWL at 6 months was 4.8% (95% CI, 2.7% to 7.0%; *P* < .001) in the FMT group and 4.6% (95% CI, 1.5% to 7.6%; *P* = .006) in the placebo group, but no difference was observed between the groups. At 18 months from the baseline (ie, 12 months after surgery), the percentage of TWL was 25.3% (95% CI, 19.5 to 31.1; *P* < .001) in the FMT group and 25.2% (95% CI, 20.2 to 30.3; *P* < .001) in the placebo group; however, no difference was observed between the groups.

**Conclusions and Relevance:**

FMT did not affect presurgical and postsurgical weight loss. Further studies are needed to elucidate the possible role of FMT in obesity.

**Trial Registration:**

ClinicalTrials.gov Identifier: NCT03391817

## Key Points

**Question:**

Does fecal microbiota transplantation contribute to weight loss in patients undergoing bariatric surgery?

**Findings:**

In this randomized clinical trial, including 41 patients treated at 2 bariatric surgery centers in Finland, no significant differences in weight loss were observed between patients receiving a fecal microbiota transplant from a lean donor vs their own fecal microbiota 6 months before obesity surgery. The surgery reduced weight equally in both groups.

**Meaning:**

In this study, fecal microbiota transplantation did not reduce the body weight of patients undergoing bariatric surgery.

## Teaser

This randomized clinical trial evaluates the effectiveness of fecal microbiota transplantation for weight loss in patients with obesity undergoing bariatric surgery.

## Introduction

Obesity is an increasingly prevalent global health concern.^1,2^ The proportion of individuals with severe obesity^1^ is rapidly increasing, and this condition is associated with a wide range of comorbidities, decreased quality of life (QoL), and increased mortality.^3^ Despite progress in behavioral and medical therapies, obesity surgery remains the most effective strategy to treat severe obesity.^3^ Bariatric surgery reduces mortality^4,5^ and increases QoL.^6^ However, a portion of patients achieves only minimal weight reduction after bariatric surgery or regains weight after initially proper weight reduction.^7,8^

The intestinal microbiota has aroused interest as a potential target for the treatment of obesity.^9^ Fecal microbiota transplantation (FMT) has been effective in treating obesity in mouse models.^10^ The differences in the intestinal microbiota of lean and obese individuals^11^ and established causality between the intestinal microbiota and body weight in animal models^10^ have fostered research on FMT for obesity and compromised metabolism^12,13,14,15,16,17,18^ and have resulted in slight improvements in insulin sensitivity,^12,14^ abdominal adiposity,^13^ and lipid metabolism^15^ but have had less effect on body weight to date.^13,16,18^ The benefits attained appear to be transient,^17^ despite successful microbial engraftment.^18^ Most patients with severe obesity harbor an intestinal microbiota with decreased bacterial diversity and microbial gene richness compared with healthy controls,^11,19^ but bariatric surgery improves microbial gene richness.^19^ We performed this placebo-controlled randomized clinical trial to investigate the effect of enriching the intestinal microbiota with FMT on the outcomes of bariatric surgery.

## Methods

### Trial Design

The study participants were recruited from Helsinki University Central Hospital, Helsinki, and Päijät-Häme Central Hospital, Lahti, Finland. Recruitment began in January 2018, and the follow-up of the last patient was completed in March 2021. The trial protocol (Supplement 1) was approved by the ethical committee of Helsinki and Uusimaa Hospital District, and written informed consent was obtained from all study participants. This study followed the Consolidated Standards of Reporting Trials (CONSORT) reporting guideline.

**Figure 1.**
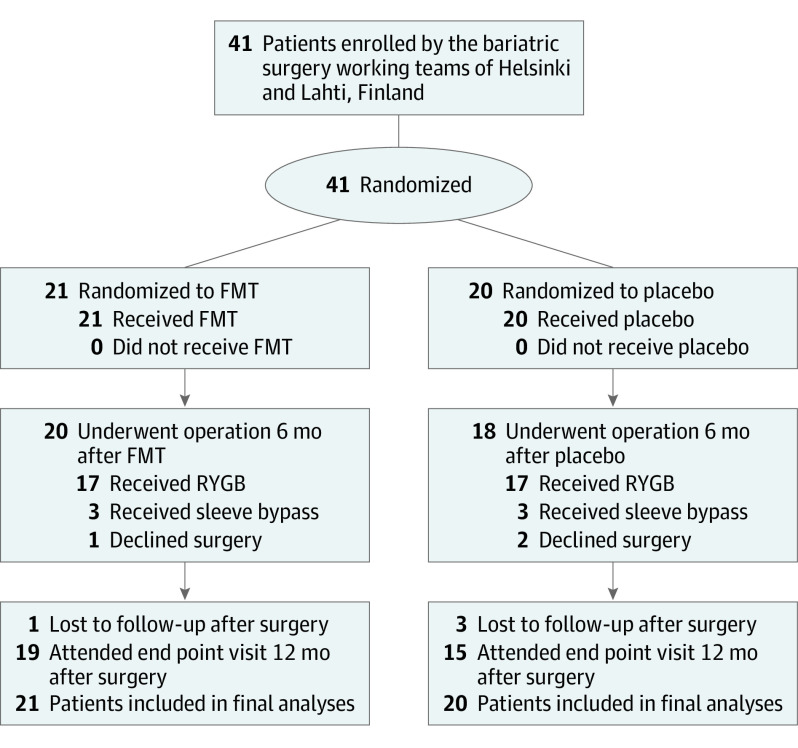
Trial Flowchart FMT indicates fecal microbiota transplantation; RYGB, Roux-en-Y gastric bypass.

We randomized 41 adult patients with obesity 1:1 to receive either FMT from a healthy lean donor or autologous placebo by gastroscopy into the duodenum. All randomized patients received the baseline intervention. All patients in the study were scheduled for obesity surgery 6 months after the baseline intervention, but 3 patients changed their mind and declined the surgery. In addition, 4 participants did not attend the final follow-up visit: 1 patient became pregnant soon after bariatric surgery, 1 declined to visit the hospital due to fear of SARS-CoV-2 infection, and 2 participants did not provide any reason for nonattendance. Thus, 19 patients in the FMT group and 15 patients in the placebo group attended the final visit at 12 months after surgery (Figure 1).

A frozen and thawed solution of donor feces (30 g in a 170 mL final volume of saline and 10% glycerol) was prepared and administered as previously described.^20^ The randomization was revealed to patients after their last visit. The endoscopists performing the baseline intervention and personnel treating the patient remained blinded until the end of follow-up of all the patients.

All patients consumed a very low-calorie diet for approximately 4 weeks preceding the surgery. As a prophylactic antibiotic, the patients who were treated in Helsinki received 1 dose of 2 g of amoxicillin orally, and patients who were treated in Lahti received a dose of 1.5 g of cefuroxime intravenously before the operation.

The study visits before obesity surgery were at baseline and 2, 4, and 6 months after baseline. The follow-up time points after surgery were 3, 6, and 12 months (ie, 1.5 years after the baseline intervention). At each of the 7 time points, blood and stool samples were obtained, weight and body composition were measured by performing a bioelectrical impedance analysis (BIA) (Tanita MC-980, Tanita Corporation), and the patients completed QoL questionnaires.

Candidates for obesity surgery were recruited to participate in this study, and the participants fulfilled the following criteria for bariatric surgery^3^: a body mass index (BMI; weight in kilograms divided by height in meters squared) greater than 40 or greater than 35 if the patient had obesity-related comorbidities, such as type 2 diabetes, hypertension, sleep apnea, nonalcoholic fatty liver disease, osteoarthritis, or hyperlipidemia. A large hiatal hernia was predefined as an exclusion criterion to prevent reflux of the FMT. Other exclusion criteria were pregnancy, type 1 diabetes, severe kidney insufficiency, and chronic or recurrent bacterial infection requiring antimicrobial treatment.

### Donors

Fecal transplants from 2 constantly lean donors were used in this study: a donor in their 40s who practiced long-distance training (BMI <20), and a donor in their 50s who was an organic gardener (BMI <25). Both donors were generally healthy without diagnosed chronic diseases or medications, and they had not used antibiotics within the preceding 12 months. They had a healthy lifestyle and were omnivores (ie, their diets included some animal products but were rich in vegetables). The donors were screened according to international guidelines to exclude communicable diseases, as well as immunological and metabolic diseases.^21^

### Outcomes

The primary outcome of the study was a reduction in body weight, which is reported as the change in BMI compared with the baseline, the percentage of total weight loss (TWL), and the percentage of excess BMI loss (EBMIL).^22^ Secondary outcomes included body composition measured with BIA, blood chemistry, and QoL. Disease-specific QoL was measured with the Moorehead-Ardelt QoL questionnaire.^23^ Health-related QoL was assessed with the 15-dimension (15D) questionnaire.^24^

### Sample Size

When planning this trial, studies assessing the effect of FMT on obesity in human participants were not published. The sample size was calculated according to the estimation that 40% of participants in the FMT group and 10% in the placebo group would reach a weight reduction of 10% by week 24. The calculated sample size was 40 patients; therefore, 20 patients were selected for both groups. The CI was selected to be 95% (α = .05 and β = 0.1).

### Statistical Analysis

We applied SPSS statistical software version 27 (IBM Statistics) to perform statistical analyses for this trial. The results are shown as the means and SD or 95% CIs for continuous variables and as numbers and percentages for dichotomous variables. A 2-sided *t* test was applied for continuous data, and the χ^2^ test was applied for nominal data. Variance of weight loss was analyzed with Levene F test.

The estimated marginal means were calculated for all variables using repeated measures analysis of variance, and Bonferroni adjustment was applied to calculate significance. *P* < .05 was considered a significant difference for all analyses. Data were analyzed from March 2021 to May 2022.

## Results

### Patient Characteristics

Forty-one patients (mean [SD] age, 48.7 [8.7] years; mean [SD] baseline BMI, 42.1 [6.0]; 29 women [70.7%]) were randomized into the FMT group (21 participants) and the placebo group (20 participants) (Figure 1). The BMI of the FMT group was higher than that of the placebo group, but the difference was not significant. Age, sex, and main comorbidities were evenly distributed across the groups. At baseline, a difference in the general QoL was observed favoring the FMT group; the mean (SD) 15D total score was 0.90 (0.07) for the FMT group and 0.81 (0.13) for the placebo group (*P* = .02) (Table 1).

**Table 1.** Patient Characteristics

Characteristic	Patients, No. (%	
	Fecal microbiota transplant	Placebo
Age, mean (SD), y	49.7 (7.1)	47.1 (10.1)
Sex		
Male	7 (33.3)	5 (25.0)
Female	14 (66.7)	15 (75.0)
Weight, mean (SD), kg	124.8 (19.6)	120.4 (23.5)
Height, mean (SD), cm	170 (0.1)	168 (0.1)
Body mass index, mean (SD)^a^	43.3 (6.0)	41.1 (5.9)
Type 2 diabetes	6 (31.6)	5 (26.3)
Dyslipidemia	4 (21.1)	2 (10.5)
Hypertension	10 (52.6)	12 (63.2)
Moorehead-Ardelt quality of life total score, mean (SD)	0.81 (0.78)	0.23 (1.01)
15-Dimension quality of life questionnaire total score, mean (SD)	0.90 (0.07)	0.81 (0.13)

^a^ Body mass index is calculated as weight in kilograms divided by height in meters squared.

### Primary End Point: Weight Reduction

**Figure 2.**
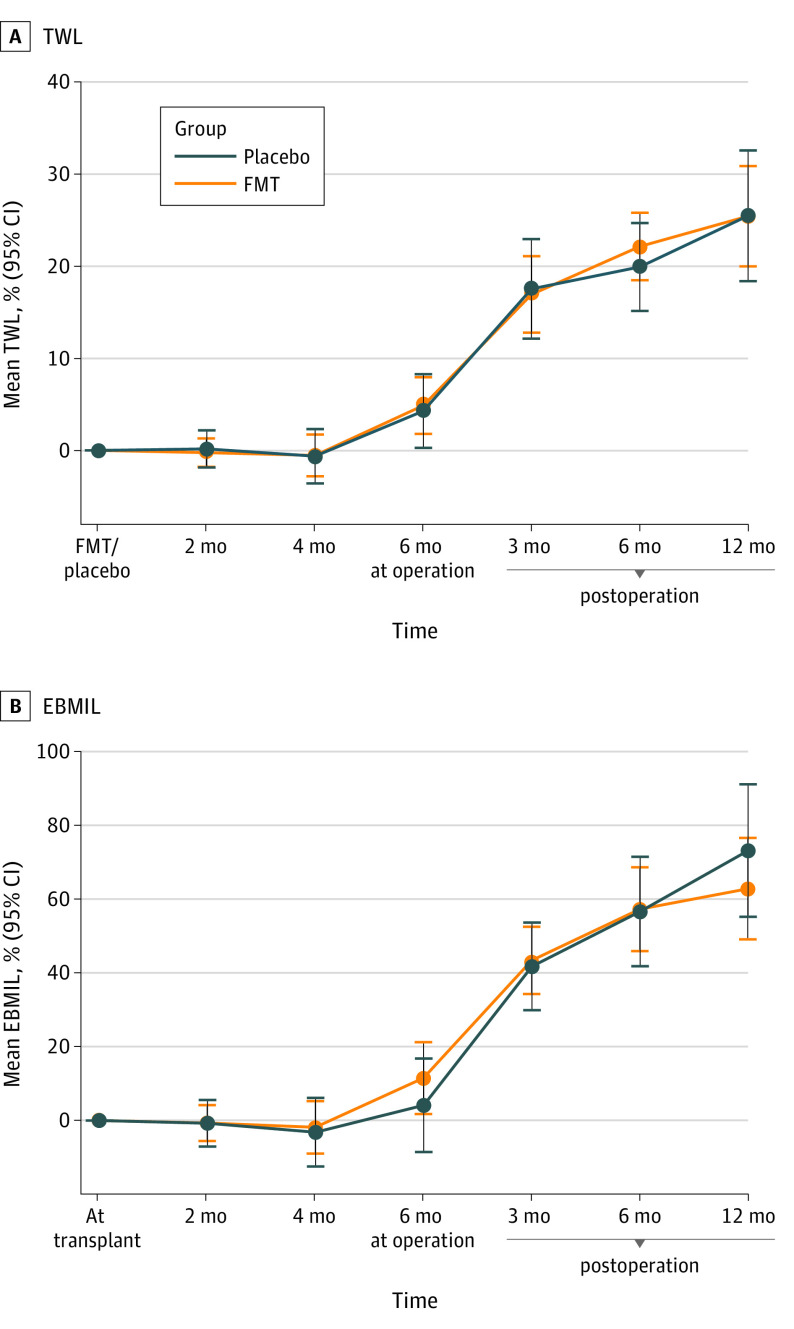
Percentage of Excess Body Mass Index Loss (EBMIL) at Different Time Points FMT indicates fecal microbiota transplantation.

The estimated marginal mean values for percentage of EBMIL (Figure 2) and all other primary end point variables (eFigure 1 in Supplement 2) were determined after considering all the measurement points.

The reduction in the mean BMI in the FMT group from baseline to the end of follow-up was 10.4 (95% CI, 8.2 to 12.5; *P* < .001). In the placebo group, the change in BMI from baseline to the end of follow-up was 10.15 (95% CI, 7.8 to 12.5; *P* < .001). The BMI at 6 months decreased 2.1 (95% CI, 1.2 to 3.1; *P* < .001) in the FMT group and 1.3 (95% CI, −0.3 to 2.9; *P* = .11) in the placebo group compared with the baseline.

The percentage of total weight loss from baseline to the 2- and 4-month time points was not significant in either study group. However, percentage of TWL from baseline to the 6-month time point was significant in both groups: 4.8% (95% CI, 7.0% to 2.7%; *P* < .001) in the FMT group and 4.6% (95% CI, 7.6% to 1.5%; *P* = .006) in the placebo group, but no difference was observed between the groups (95% CI, −3.8% to 3.3%; *P* = .89). From baseline to the end of follow-up, the mean weight was reduced by 25.3% (95% CI, 31.1% to 19.5%; *P* < .001) in the FMT group and 25.2% (95% CI, 30.3% to 20.2%; *P* < .001) in the placebo group; however, no difference was observed between the groups (Table 2).

**Table 2.** Percentage of TWL at Different Time Points

Time point	TWL, mean (SD), %		*P* value
	Fecal microbiota transplant	Placebo	
Time after baseline, mo			
2	−0.2 (2.1)	−0.3 (3.3)	.90
4	0.1 (3.5)	−0.0 (5.1)	.90
6	4.8 (4.6)	4.6 (6.1)	.89
Time after surgery, mo			
3	16.3 (6.1)	16.6 (9.1)	.88
6	21.5 (8.2)	19.9 (6.2)	.53
12	25.3 (12.0)	25.2 (9.1)	.99

Abbreviation: TWL, total weight loss.

The percentage of EBMIL from baseline to the end of follow-up was comparable between the groups: 62.9% (95% CI, 77.6%-48.2%; *P* < .001) in the FMT group and 69.4% (95% CI, 81.5%-57.4%; *P* < .001) in the placebo group. The operation type did not alter the result (eFigure 2 in Supplement 2). No significant differences in change in BMI, percentage of EBMIL, or percentage of TWL were observed at any time points between the groups. The variance in percentage of TWL at 12 months after surgery was not significantly different between the FMT group and the placebo group (SD^2^ 144.0 vs 82.5).

### Body Composition

From baseline to 4 months, the mean (SD) fat percentage increased by 0.2% (1.5%; 95% CI, −0.6% to 1.0%; *P* = .58) in the FMT group and 2.2% (4.1%; 95% CI, 0.1% to 4.4%; *P* = .05) in the placebo group. Mean (SD) visceral fat increased by 0.1% (1.2%; 95% CI, −0.5% to 0.7 %; *P* = .71) in the FMT group and 0.6% (1.7%; 95% CI, −0.2% to 1.5%; *P* = .14) in the placebo group. The mean (SD) muscle mass increased by 0.2 (1.5) kg (95% CI, −0.9kg to 0.6; *P* = .66) in the FMT group and 2.5 (5.4) kg (95% CI, −5.3kg to 0.2; *P* = .07) in the placebo group (eTable 1 in Supplement 2).

From baseline to the end of follow-up at 12 months after surgery, the mean (SD) fat percentage decreased by 8.8% (7.3%; 95% CI, 5.1% to 12.3%; *P* < .001) in the FMT group and 7.6% (5.2%; 95% CI, 4.8% to 10.6%; *P* < .001) in the placebo group. The mean (SD) visceral fat content decreased by 6.9% (2.4%; 95% CI, 4.9% to 8.8.%; *P* < .001) in the FMT group and 5.3% (3.9%; 95% CI, 4.0% to 6.7%; *P* < .001) in the placebo group. The mean (SD) muscle mass decreased 8.0 (3.3) kg (95% CI, 6.3 to 9.8; *P* < .001) in the FMT group and 9.9 (7.9) kg (95% CI, 5.5 to 14.2; *P* < .001) in the placebo group (eTable 1 in Supplement 2). Significant differences were not observed between the groups.

### Blood Chemistry

From the baseline intervention to 4 months, the mean (SD) cholesterol level in the FMT group decreased to 84.6 (12.6) mg/dL (*P* = .21) and increased in the placebo group to 91.8 (14.4) mg/dL (*P* = .62), and the difference between the groups was significant (*P* = .02) (to convert to millimoles per liter, multiply by 0.0259). No other significant differences in the laboratory test results were observed between the groups. The mean values of all obtained laboratory tests at every measurement point are presented in eTable 2 in Supplement 2.

From baseline to the end of follow-up, the mean high-density lipoprotein cholesterol level increased by 5.4 mg/dL (95% CI, 1.8 to 7.2; *P* < .001) and 5.4 mg/dL; (95% CI, 3.6 to 7.2; *P* < .001) in the FMT group and the placebo group, respectively, while the mean low-density lipoprotein cholesterol levels decreased by 3.6 mg/dL (95% CI, −3.6 to 10.8; *P* = .26) and 9 mg/dL; (95% CI, 3.6 to 14.4; *P* = .003) in the FMT group and the placebo group, respectively. The mean triglyceride level decreased by 12.6 mg/dL (95% CI, 1.8 to 23.4; *P* = .03) and 10.8 mg/dL (95% CI, −0.0 to 16.2; *P* = .06) in the FMT group and the placebo group, respectively. Fasting glucose levels decreased by 20.52 mg/dL (95% CI, 1.08 to 39.96; *P* = .04) in the FMT group and 12.25 mg/dL (95% CI, 12.24 to 21.24; *P* = .01) in the placebo group (to convert to millimoles per liter, multiply by 0.0555). Hemoglobin A_1c_ (HbA_1c_) levels decreased to 2.8% (95% CI, 2.3% to 3.3%; *P* = .14) and 2.7% ; (95% CI, 2.2% to 3.2%; *P* = .02) in the FMT group and the placebo group, respectively (to convert to proportion of total hemoglobin, multiply by 0.01). Uric acid levels decreased in the FMT group by 0.96 mg/dL (95% CI, 0.47 to 1.45; *P* = .001) and in the placebo group by 0.16 mg/dL (95% CI, −0.63 to 0.94; *P* = .67) (to convert to millimoles per liter, multiply by 0.0595). However, from surgery to the end of follow-up, the mean uric acid level also decreased in the placebo group to 1.09 mg/dL (95% CI, 0.46 to 1.72; *P* = .002). No significant differences in the estimated marginal mean values of lipid, HbA_1c_, or uric acid levels were observed (eFigure 3 in Supplement 2).

### Quality of Life

#### 15D: the General Quality of Life

The estimated marginal mean values for the 15D total score considering all the measurement points were not significantly different between the groups (eFigure 4 in Supplement 2). When the mean total score of the 15D at baseline was compared with the scores of the subsequent measurement points, the only significant changes were the increases in the placebo group at 4 months of 0.044 (95% CI, 0.001-0.088; *P* = .046) and 6 months of 0.054 (95% CI, 0.006-0.102; *P* = .03) and 3 months postoperatively of 0.066 (95% CI, 0.015-0.116; *P* = .01); these changes were also clinically significant.^25^

#### Moorehead-Ardelt Quality of Life: the Disease-Specific Quality of Life

**Figure 3.**
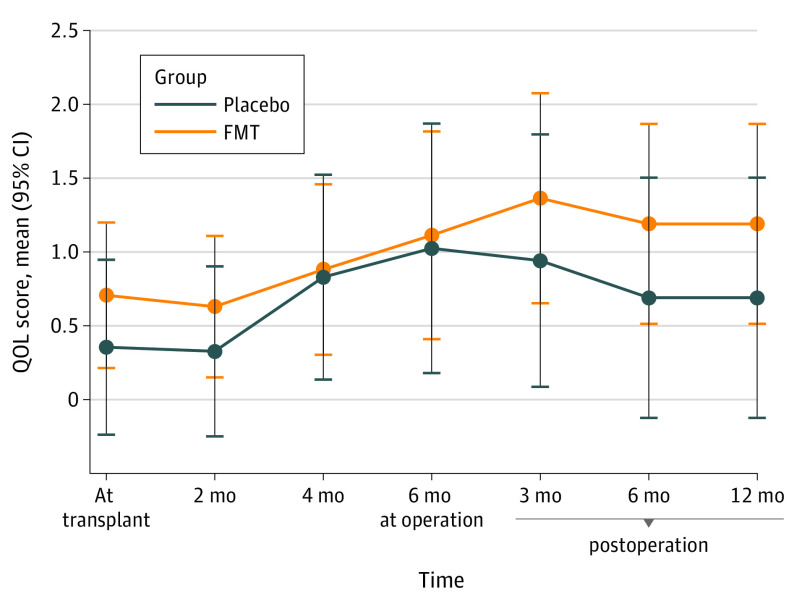
Moorehead-Ardelt Quality of Life (QoL) FMT indicates fecal microbiota transplantation.

The estimated marginal mean values of the Moorehead-Ardelt QoL total score considering all measured time points were not significantly different between the groups (Figure 3). As the baseline value of the mean Moorehead-Ardelt QoL score was compared with the subsequent time points, significant increases were observed at the time points of 4 months 0.45 (95% CI, 0.07-0.86; *P* = .02) and 6 months 0.86 (95% CI, 0.24-1.48; *P* = .01) in the placebo group and at the time points of 4 months 0.3 (95% CI, 0.02-0.58) and 6 months after the baseline 0.55 (95% CI, 0.86-0.23; *P* = .03) as well as at 3, 6, and 12 months after the surgery in the FMT group.

### Adverse Events

No complications related to the FMT procedures were reported. The patients were not under deep sedation, the transplant was infused deep into the duodenum, and the patients were asked to return to upright position after the procedure to prevent reflux of the fecal transplant. No serious adverse events related to the baseline intervention or bariatric surgery occurred in either group.

## Discussion

In this placebo-controlled, randomized clinical trial for obesity, significant changes in body weight were not observed in the 4 months after FMT or placebo administration. As expected, bariatric surgery 6 months after the baseline intervention reduced weight in both groups. From baseline to 6 months, as a result of the preoperative course of very low-calorie diet, percentage of TWL was 4.7%, and 1 year after the operation, the mean body weight of the whole study group was 25.3% lower than at the baseline without any differences between the groups.

Our study provides further evidence that FMT alone is not sufficient to decrease body weight in humans. However, FMT may exert a transient effect on more delicate markers of metabolism.^12,17^

A desirable goal is to develop a method to reduce the variation in the weight loss outcomes of obesity surgery. In our trial, more variance in the postsurgical body weight loss was observed in the placebo group than in the FMT group; however, the difference was not significant.

A review^26^ of previous trials on FMT for metabolic syndrome concluded that patients presented a reduction in HbA_1c_ levels and an increase in HDL levels 2 to 6 weeks after FMT; however, our trial did not detect any similar changes at the 2-month time point. Some of the changes mediated by FMT may have lasted for such a short period that our first measurement point at 2 months was set too late to detect those changes. The blood urate level increased after the placebo treatment but not after FMT, but urate levels decreased in both groups after surgery. Bariatric surgery is known to reduce blood urate levels in the long term, but in the short term, it results in a drastic fluctuation in blood urate levels, predisposing the patient to a gout flare.^27^

We recorded a reduction in muscle mass after bariatric surgery. Preliminary evidence suggests that decreases in perioperative muscle mass do not result in a loss of muscle strength.^28^ In the cohort analyzed by Leong and colleagues,^13^ abdominal adiposity was lower after FMT, as measured using dual-energy x-ray absorptiometry. Our results do not support this finding, as the visceral fat content increased marginally at 2 and 4 months after FMT and the placebo treatment. As expected, the visceral fat content was reduced postoperatively in both groups. Although we applied BIA instead of dual-energy x-ray absorptiometry, the use of different methods does not explain the discrepancy in the results, as the methods have a good correlation in evaluating body composition among individuals with severe obesity.^29^

This trial had a longer follow-up time than that reported in most of the FMT studies conducted to date.^30^ The study participants constituted a homogeneous cohort. The morbidity of obesity of the patients undergoing bariatric surgery is well defined, and the participants were in close surveillance by dietitians through the follow-up.

### Strengths and Limitations

The use of an autologous feces-based placebo enabled reliable blinding; however, the gut microbiota may change immediately after defecation even when oxygen exposure is short. In addition, colon-derived microbiota may change the local microenvironment in the small intestine and affect the ample and diverse immune system of the small bowel. Thus, autologous FMT is not an inert placebo when administered via the upper route.^31,32^ The perioperative antibiotics, amoxicilline and cefuroxime, may have had different impacts on gut microbiota.

The main limitation of our study is the number of patients, which may be inadequate to show possible minor effects of FMT on weight. In the absence of published data, our estimation of the treatment effect was optimistic, and, thus, the number of patients was tuned to detect only clear differences between the groups. Limited numbers of patients may generate a type II error, and we were unable to determine whether a much larger sample size would have yielded any differences between the groups according to these results.

## Conclusions

In this randomized clinical trial, FMT by gastroscopy into the duodenum did not affect the body weight of participants with obesity. Bariatric surgery 6 months after FMT or placebo administration reduced weight equally in both groups during the 1-year follow-up. No major adverse events related to either intervention were recorded.

## Notes

Supplement 1.
Trial Protocol

Supplement 2. **eFigure 1. **Main End Points **eFigure 2. **Estimated Marginal Means of %EBMIL by Operation Type **eTable 1. **Body Composition **eTable 2. **Blood Chemistry **eFigure 3. **Blood Chemistry: Variables Associated With Metabolism **eFigure 4. **15D Quality of Life

Supplement 3.
Data Sharing Statement
