# Supplementary material for: Effectiveness of Fecal Microbiota Transplantation for Weight Loss in Patients With Obesity Undergoing Bariatric Surgery: A Randomized Clinical Trial
Source: JAMA Netw Open. 2022 Dec 16;5(12):e2247226. doi: 10.1001/jamanetworkopen.2022.47226 (PMC9856235; doi:10.1001/jamanetworkopen.2022.47226)
Supplement: Supplement 1. — Trial Protocol [file jamanetwopen-e2247226-s001.pdf]

1

Title of the project

FECAL MICROBIOTA TRANSPLANTATION IN THE TREATMENT OF MORBID OBESITY

Study group:
Perttu Arkkila Ph.D. M.D. Department of Gastroenterology, Helsinki University Hospital
Perttu Lahtinen M.D. Department of Gastroenterology, Central Hospital Lahti
Anne Juuti M.D. Department of GI-surgery, Helsinki University Central Hospital
Eero Mattila M.D. Department of Infectious Disease, Helsinki University Hospital
Reetta Satokari, Ph.D. Department of Bacteriology and Immunology, Faculty of Medicine, University of Helsinki, Helsinki, Finland
Veli-Jukka Anttila Ph.D. M.D. Department of Infectious Disease, Helsinki University Hospital
Markku Luostarinen Ph.D. M.D. Department of Gastrosurgery, Central Hospital Lahti

Personal and contact Information

Name	Perttu Arkkila
Current Work	Associate Chief Physician
Address	PL 340, 00290 Helsinki, Finland
Phone Nr.	+358 50 4272272
E-mail	perttu.arkkila@hus.fi

Background

Obesity is one of the biggest public health issues, especially in the Western world. The prevalence of obesity has increased in most parts of the world over the last decades (Finucane 2011). Over 33% of the world's adult population are overweight or obese; in future this number is estimated to be as high as 57.8% (WHO 2014). The Prevalence of obesity among adults (BMI >30kg/m2) in Finland was 21% in a 2007 survey (Vartiainen). In the USA, the age-adjusted prevalence of obesity from 2009-2010 was 35,5% in adult men and 35,8% in adult women (Flegal 2012). Obesity has recently been identified as a disease by the American Medical Association (AMA).

Obesity is an important risk factor for diabetes, elevated blood pressure, cardiovascular disease, cancer and overall mortality. The lifespan of severely obese individuals is estimated to shorten by 5-20 years (Whitlock, Fontaine). Weight loss is associated with improvement of the components of metabolic syndrome and weight loss achieved by bariatric surgery reduces mortality (Sjöström)

Each individual has their own unique gut microbiome which is affected by genome, microbial exposure, age, diet, environment and geographical location (The Human Microbiome Consortium 2012, Ursell 2012, Yatsusenko 2012, Jayasinghe 2016). Four bacterial phyla form the vast majority

2

of gut microbiome of a healthy individual: Firmicutes, Bacteroidetes, Proteobacteria and Actinobacteria. Typically, approximately 90% of fecal bacteria is either gram positive Bacteroidetes or gram negative Firmicutes. Obesity is associated with a change in gut microbiome. The gut microbial composition of an obese individual tends to show reduced complexity (Turnbaugh 2009). An increase in relative abundance of Firmicutes and a proportional decrease in Bacteroidetes has been detected in studies of obese mice and men (Ley 2005 and 2006). A 20% increase in Firmicutes and a corresponding 20% decrease in Bacteroidetes is estimated to provide an additional 150kcal of energy per day (Jumpertz 2011). It is possible though that microbial changes associated with obesity are not simply phylum based but rather the result of a collection of numerous small differences within the overall population structure (Walters 2014). It seems likely that individuals at high risk to develop obesity have quantitative and qualitative differences in gut microbiota compared with individuals at low obesity risk (Diamant 2010). The composition of gut microbiome predicts the response to different dietary interventions in obese individuals (Korpela 2014, Lappi 2013).

The causal relationship of microbiome and obesity in humans has not yet been demonstrated, but in a study with germ-free mice, FMT with “obesogenic microbiota” caused more body fat accumulation compared to FMT with “a lean microbiota” (Turnbaugh 2006). Despite encouraging evidence, randomized controlled studies of FMT in obesity or metabolic disease are scarce. To date only one such comprehensive study has been published, in which gut microbiota transplanted from lean donors to individuals with metabolic syndrome significantly increased their insulin sensitivity (Vrieze 2012). In the study, 9 middle-aged men with metabolic syndrome were treated with FMT; the size of the control group was 9 individuals. The follow-up time in the study was only 6 weeks which, it has been argued, was too short to demonstrate possible changes in weight (Jayasinghe 2016).

Treatment of recurrent *Clostridium difficile*-infection (CDI) with antibiotics leads to recurrences in up to 50% of patients. Our study group has recently shown that fecal microbiota transplantation (FMT) through colonoscopy was an effective treatment for recurrent CDI in over 90% of CDI patients. Transplantation was done for 70 patients and no significant complications arose from the procedure (Mattila 2012). FMT is nowadays used as a routine procedure in clinical practice in treatment of patients with recurrent CDI.

It has not been clear to what extend FMT transfers a new microbiota or just modulates the existing microbiota. (Jayasinghe 2016). New studies have shown the durable coexistence of donor and recipient strains after fecal microbiota transplantation (Li 2016, Jalanka 2016 submitted). In our

3

study on recurrent CDI, we showed long-term (1 year) and stable engraftment of that donor´s microbiota in the patients (Jalanka et al, submitted). Whether such a stable engraftment of donor´s microbiota exists in other patient groups remains to be seen. Further, our study revealed specific bacterial taxa that were commonly established in all CDI patients (but were absent before the treatment), i.e. a therapeutic core microbiota consisting of 24 bacterial taxa (Jalanka et al,submitted).This is promising in relation to the possible development of bacteriotherapy based on pure cultures of bacteria in the future. Overall, the recent scientific and clinical results have highlighted the potential benefits that obese patients could gain through treatmentby FMT using stool from a lean donor. The efficacy and safety of this treatment should be studied in a double blind study.

The aim of our study is to investigate the efficacy and safety of fecal transplantation in treatment of obesity. Another aim is to analyse fecal microbiota in order to find micro-organisms contributing to clinical outcomes in fecal transplantation in obese patients.

STUDY DESIGN

STUDY POPULATION

Inclusion criteria for study

- Candidates for the bariatric surgery
    - BMI ≥ 40 or BMI ≥35 and at least two obesity-related co-morbidities such as type II diabetes (T2DM), hypertension, sleep apnea and other respiratory disorders, non-alcoholic fatty liver disease, osteoarthritis, lipid abnormalities, gastrointestinal disorders, or heart disease.
- Availability of consecutive fecal samples during one year
- Compliance to attend gastroscopy and FMT
- 18-65 years

Exclusion criteria for study

- Unable to provide informed consent
- Pregnancy
- Type I Diabetes Mellitus

4

- Severe renal insufficiency, GFR<30%
- Chronic or recurrent bacterial infection needing antimicrobial treatment
- Large hiatal hernia

METHODS

40 consecutive adult obese patients filling the criterion for bariatric surgery will be recruited. Patients are recruited from Helsinki University Central Hospital and Päijät-Häme Central Hospital. Fecal microbiota transplantation or FMT with own feces will be administered in the gastroscopy that is a routine procedure prior to bariatric surgery. FMT is performed by experienced endoscopists using frozen and thawed stool (for the protocol of fecal banking, see Satokari et al 2015) through a gastroscope into the duodenum. 20 patients will receive a fecal transplantation from a lean healthy tested donor and 20 patients in the control group will receive their own feces donated prior to the gastroscopy. The endoscopists and personnel performing the FMT are blinded for the type of feces. The randomization is done by a doctor not attending the study. Bariatric surgery will be carried out at week 24 after FMT. Weight, waist circumference, blood pressure, blood and stool samples will be obtained at week 6, 12, 24 and 48. Blood samples include the same metabolic parameters ***(hemoglobin, lipid and glucose status)*** which belong to the routine clinical management of bariatric surgery patients. In addition to this, blood samples ***(four tubes, max 20 ml blood)*** are collected for further evaluation of possible new metabolic and inflammation parameters. The examinations on week 24 will be done before the surgery.

Study protocol in details:

1. ***Evaluation of the patients by endocrinologist .***
2. ***Preoperative gastroscopy to be sure that there are no contraindications for bariatric surgery.***
3. ***Fecal microbiota transplantation (healthy lean donor, whose stool is carefully tested and infective diseases are excluded by lab values).***
4. ***Interview of side effects (infections, dyspeptic or other gastrointestinal symptoms, any other adverse events) are done at week 0, 6, 12, 24, 36 and 48.***
5. ***GERD- and quality of life questionnaires before gastroscopy and 24 and 48 weeks after gastroscopy.***
6. ***Measurement of weight at week 0, 6, 12, 24, 36 and 48.***

5

Follow-up of the patients includes an interview and examination at week 0, 6, 12, 24, 36 and 48. A general quality of life measurement (15D-questionnaire, www.15d-instrument.net), and GERD questionnaire (GERD-Q-questionnaire) is done at the day of stool transplantation, 24 weeks after the transplantation and 24 weeks after surgery, i.e., 1 year after FMT. After the study, the patients are screened again at 2 years and 5 years for long term efficacy and safety. Any adverse events are recorded. Changes in the use of medication will be recorded.

DONOR SELECTION

Stool donors are generally unfamiliar to the patient. Stool is provided from the stool bank of the hospital. Donor testing has been described in articles by Mattila et al 2012 and Arkkila et al 2013. Related or familiar donors are accepted, if desired by the patient. The donors are lean (BMI <25) generally healthy adults.

Donors are excluded if they have used antibiotics within the preceding 12 months; are on immunosuppressive or chemotherapeutic agents; have known or recent exposure to HIV; hepatitis B or C; have a current communicable disease; are obese; have IBD, IBS, atopy, chronic diarrhea or constipation; GI malignancy or polyposis; have depression, anxiety, autism or other diagnosed psychiatric condition, have multiple sclerosis or Parkinson disease; have excessive use of alcohol; participate in high-risk sexual behaviors; use illicit drugs; have a history of recent incarceration or travel to areas with endemic diarrhea. Donor blood testing includes tests for HIV, hepatitis A, B and C; donor stool testing includes culture C. difficile toxin, ova and parasites, and Helicobacter pylori.

6

Figure 1. Study schema for the study of fecal transplantation in obesity

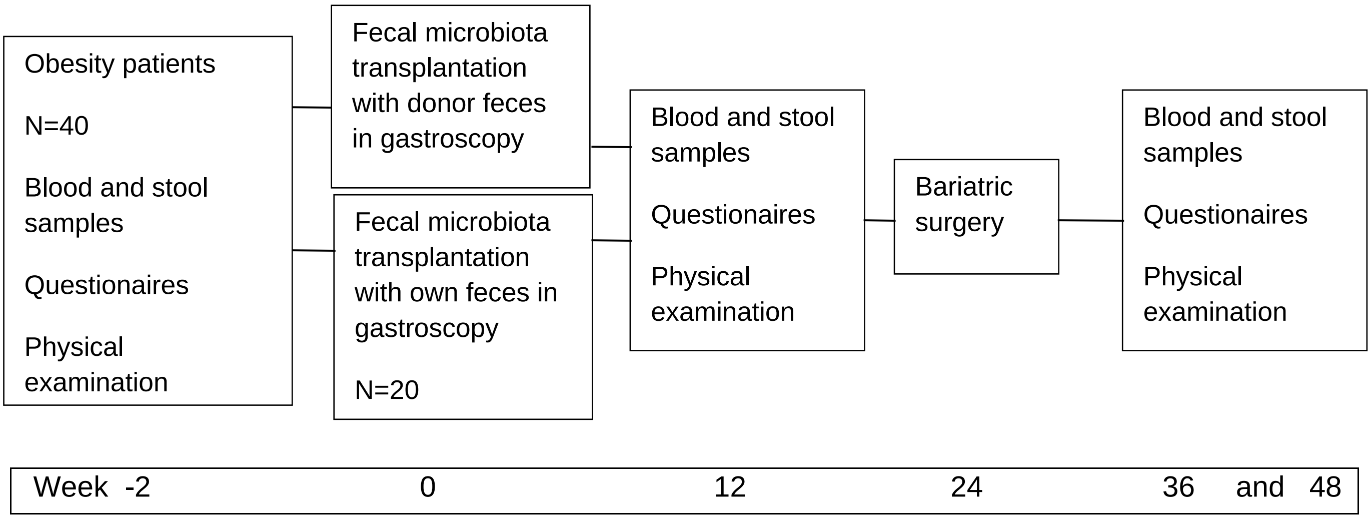

The endpoint of the study is a reduction of weight at 24 ad 48 weeks. Secondary endpoints include reduction of blood glucose levels, cholesterol levels and blood pressure. As a secondary objective for this study we will evaluate the changes in gut microbiota within the 48 weeks. We also aim to estimate more subtle changes reflecting the severity of the metabolic syndrome, such as obesity associated hormones, inflammation parameters, and metabolomics.

Microbiota analysis

Microbiota analysis will be done by Docent Reetta Satokari´s research group, who have a solid track record in the area of intestinal microbiota research (for publications, see Satokari's publicly available GoogleScholar profile). Briefly, microbiota profiling will be performed by high-throughput sequencing of the V1-V3 variable region of the 16S rRNA gene by using the Illumina MiSeq platform according to the manufacturer´s specifications, and generating paired-end reads of 300 bp in length in each direction and 50 bp overlap, with the final read length of 550 bp. The sequencing will be done in the core facility of the University of Helsinki. The 16S rDNA sequence data will be processed by using the QIIME (Quantitative Insights Into Microbial Ecology) pipeline, which is an open-source bioinformatics software package designed for microbial community analysis based on DNA sequence data and by using in-house analysis pipelines. Once obtained, the sequences will be curated and assigned to operational taxonomic units (OTUs), which are considered as approx.

7

equivalents to bacterial species. The main focus of the analysis is on the similarity of the recipients´ microbiota to that of the donor and the stability of microbiota post-FMT and the differences in microbiota characteristics between the two FMT–regime groups and controls. In addition, the universal donor approach of the study will allow a controlled analysis of bacterial taxa engrafted in all patients receiving donor´s microbiota and the aim is to also pin-point specific bacterial taxa that are associated with the clinical outcome.

STATISTICAL ANALYSIS

The sample size is calculated according to the estimation that the difference in weight reduction of 10 % in week 24 is 40% in the group of FMT from lean donor and 10% in the control group (FMT from own feces). The calculated sample size is 40 patients and therefore 20 patients are selected for both groups (donor vs own feces). This difference is considered to be clinically meaningful. The confidence interval was selected to be 95% (α=0.05 and β=0.1).

DATE

Patient recruitment will be made from October 2016 until the end of 2018. The follow-up of the patients will end half a year after the last patient concluded the study. Data analysis and reporting of the results will be completed by the end of 2020.

BUDGET PLAN

This study includes one gastroscopy at week 0 which is a standard procedure prior to bariatric surgery and no extra expenditure appears. Other data will be collected in routine visits.

Funding is being applied from *Ehrnrooth foundation* for labor costs caused by bioinformative analyzes,such as profiling the microbiome of the patients and analyzing inflammatory and metabolic markers.

8

Wages:	Physicians	1 months	7000 euros
	Study nurse	2 months	7000 euros

Preparation of stool for the transplantation and testing 7000 euros
Microbiota analysis 9000 euros
Total **30,000 euros**

ETHICAL ASPECTS AND INFORMATION ABOUT PATIENTS' RIGHTS

The ethical risks for this study include the possible transmission of harmful microbiota to the patients, causing some infection or other diseases. The risk is most likely minimal, because the feces and the donors are widely tested for many known pathogens and diseases. All the patients are informed about the experimental nature of this treatment procedure and about the available results in previously published reports and possible risks of the procedure. All of the patients are asked for informed consent. A p***atient can refuse to attend this study and it does not affect his or her treatment thereafter. A patient can also stop the follow-up of the study at any time. There is no special insurance for this study, but patients are taken care of as any other patients after possible infectious or other complications in the hospital. All the information collected from the patients is stored on the hospital database and no information showing data of patient identification is sent or stored in other places.***

Permission for this study will be applied from the Institutional Review Board of Helsinki University Hospital.

PUBLICATION PLAN

The results of this study will be published at the international medical meetings in the fields of gastroenterology and endocrinology. Manuscripts of the study will be sent to international medical journals. Depending on the results, there will be one or several publications.

WHAT MAKES THIS RESEARCH INNOVATIVE

9

Placebo-controlled studies of fecal transplantation in obesity have not been published before. This study will provide information in relation to the question asked in many meta-analyses – can weight be reduced and metabolic syndrome relieved by manipulating the gut microbiome?

REFERENCES

Arkkila P, Mattila E, Anttila V-J. Ulosteensiirto Clostridium difficile -infektion hoitona Lääketieteellinen Aikakauskirja Duodecim 2013;129(16):1671-9.

Jayasinghe TN, Chiavaroli V, Holland DJ, Cutfield WS, O'Sullivan JM. The New Era of Treatment for Obesity and Metabolic Disorders: Evidence and Expectations for Gut Microbiome Transplantation. Front Cell Infect Microbiol. 2016 Feb 19;6:15. doi: 10.3389/fcimb.2016.00015. eCollection 2016. Review

Vartiainen E, Laatikainen T, Peltonen M, Juolevi A, Männistö S, Sundvall J, Jousilahti P, Salomaa V, Valsta L, Puska. Int J Epidemiol. 2010 Apr;39(2):504-18. doi: 10.1093/ije/dyp330. Epub 2009 Dec 3. Thirty-five-year trends in cardiovascular risk factors in Finland.

The Human Microbiome Project Consortium (2012). Structure, function and diversity of the healthy human microbiome. Nature 486, 207–214. 10.1038/nature11234

Ursell L. K., Clemente J. C., Rideout J. R., Gevers D., Caporaso J. G., Knight R. (2012). The interpersonal and intrapersonal diversity of human-associated microbiota in key body sites. J. Allergy Clin. Immunol. 129, 1204–1208. 10.1016/j.jaci.2012.03.010

Yatsunenko T., Rey F. E., Manary M. J., Trehan I., Dominguez-Bello M. G., Contreras M., et al. . (2012). Human gut microbiome viewed across age and geography. Nature 486, 222–227. 10.1038/nature11053

Jumpertz R., Le D. S., Turnbaugh P. J., Trinidad C., Bogardus C., Gordon J. I., et al. . (2011). Energy-balance studies reveal associations between gut microbes, caloric load, and nutrient absorption in humans. Am. J. Clin. Nutr. 94, 58–65. 10.3945/ajcn.110.010132

Ridaura VK. Gut microbiota from twins discordant for obesity modulate metabolism in mice.

10

Science. 2013 Sep 6;341(6150):1241214. doi: 10.1126/science.1241214.

Arora T et al. The gut microbiota and metabolic disease: current understanding and future perspectives. J Intern Med. (2016)

Turnbaugh PJ, Ley RE, Mahowald MA, Magrini V, Mardis ER, Gordon JI. An obesity-associated gut microbiome with increased capacity for energy harvest.
Nature. 2006 Dec 21;444(7122):1027-31.

Smits LP et al. Therapeutic potential of fecal microbiota transplantation. Gastroenterology. 2013 Nov;145(5):946-53. doi: 10.1053/j.gastro.2013.08.058. Epub 2013 Sep 7

Alang N1, Kelly CR2. Open Forum Infect Dis. 2015 Feb 4;2(1):ofv004. doi: 10.1093/ofid/ofv004. eCollection 2015. Weight gain after fecal microbiota transplantation.

Sjöström L Review of the key results from the Swedish Obese Subjects (SOS) trial - a prospective controlled intervention study of bariatric surgery. J Intern Med. 2013 Mar;273(3):219-34. doi: 10.1111/joim.12012. Epub 2013 Feb 8. Review.

Flegal KM, Carroll MD, Kit BK, Ogden CL. Prevalence of obesity and trends in the distribution of body mass index among US adults, 1999-2010. JAMA 2012; 307: 491–7.

Finucane MM, Stevens GA, Cowan MJ et al. National, regional, and global trends in body-mass index since 1980: systematic analysis of health examination surveys and epidemiological studies with 960 country-years and 9.1 million participants. Lancet 2011; 377: 557–67.

Whitlock G, Lewington S, Sherliker P et al. Body-mass index and cause-specific mortality in 900 000 adults: collaborative analyses of 57 prospective studies. Lancet 2009; 373: 1083–96.

Fontaine KR, Redden DT, Wang C, Westfall AO, Allison DB. Years of life lost due to obesity. JAMA 2003; 289: 187–93.

Ley RE, Turnbaugh PJ, Klein S, Gordon JI. Microbial ecology: human gut microbes associated with obesity. Nature 2006;444:1022–1023pmid:17183309

11

Turnbaugh PJ, Ley RE, Mahowald MA, Magrini V, Mardis ER, Gordon JI. An obesity-associated gut microbiome with increased capacity for energy harvest. Nature 2006;444:1027–1031

World Health Organization (2014). Global Status Report on Noncommunicable Diseases. World Health Organization; Available online at: http://apps.who.int/iris/bitstream/10665/148114/1/9789241564854_eng.pdf?ua=1.

Mattila E, Uusitalo-Seppälä R, Wuorela M, Lehtola L, Nurmi H, Ristikankare M, Moilanen V, Salminen K, Seppälä M, Mattila PS, Anttila VJ, Arkkila P. Fecal transplantation, through colonoscopy, is effective therapy for recurrent Clostridium difficile infection. Gastroenterology. 2012 Mar;142(3):490-6.

Korpela K, Flint HJ, Johnstone AM, Lappi J, Poutanen K, Dewulf E, Delzenne N, de Vos WM, Salonen A. Gut microbiota signatures predict host and microbiota responses to dietary interventions in obese individuals. PLoS One. 2014 Mar 6;9(6):e90702. doi: 10.1371/journal.pone.0090702. eCollection 2014. PubMed PMID: 24603757; PubMed Central PMCID: PMC3946202.

Lappi J, Salojärvi J, Kolehmainen M, Mykkänen H, Poutanen K, de Vos WM, Salonen A. Intake of whole-grain and fiber-rich rye bread versus refined wheat bread does not differentiate intestinal microbiota composition in Finnish adults with metabolic syndrome. J Nutr. 2013 May;143(5):648-55. doi: 10.3945/jn.112.172668. Epub 2013 Mar 20. PubMed PMID: 23514765.

Vrieze A, Van Nood E, Holleman F, Salojärvi J, Kootte RS, Bartelsman JF, Dallinga-Thie GM, Ackermans MT, Serlie MJ, Oozeer R, Derrien M, Druesne A, Van Hylckama Vlieg JE, Bloks VW, Groen AK, Heilig HG, Zoetendal EG, Stroes ES, de Vos WM, Hoekstra JB, Nieuwdorp M. Transfer of intestinal microbiota from lean donors increases insulin sensitivity in individuals with metabolic syndrome. Gastroenterology. 2012 Oct;143(4):913-6.e7. doi: 10.1053/j.gastro.2012.06.031. Epub 2012 Jun 20. Erratum in: Gastroenterology. 2013 Jan;144(1):250. PubMed PMID: 22728514.
Diamant M, Blaak EE, de Vos WM. Do nutrient-gut-microbiota interactions play a role in human obesity, insulin resistance and type 2 diabetes? Obes Rev. 2011 Apr;12(4):272-81. doi: 10.1111/j.1467-789X.2010.00797.x. Epub 2010 Aug 26. Review. PubMed PMID: 20804522.

12

Li SS, Zhu A, Benes V, Costea PI, Hercog R, Hildebrand F, Huerta-Cepas J, Nieuwdorp M, Salojärvi J, Voigt AY, Zeller G, Sunagawa S, de Vos WM, Bork P. Durable coexistence of donor and recipient strains after fecal microbiota transplantation. Science. 2016 Apr 29;352(6285):586-9. doi: 10.1126/science.aad8852. PubMed PMID: 27126044.

Satokari et al. Simple fecal preparation and efficacy of frozen inoculum in fecal microbiota transplantation for recurrent Clostridium difficile infection. Aliment Pharmacol Ther. 2015;41:46
