# Supplementary material for: Effectiveness of Fecal Microbiota Transplantation for Weight Loss in Patients With Obesity Undergoing Bariatric Surgery: A Randomized Clinical Trial
Source: JAMA Netw Open. 2022 Dec 16;5(12):e2247226. doi: 10.1001/jamanetworkopen.2022.47226 (PMC9856235; doi:10.1001/jamanetworkopen.2022.47226)
Supplement: Supplement 2. — eFigure 1. Main End Points eFigure 2. Estimated Marginal Means of %EBMIL by Operation Type eTable 1. Body Composition eTable 2. Blood Chemistry eFigure 3. Blood Chemistry: Variables Associated With Metabolism eFigure 4. 15D Quality of Life [file jamanetwopen-e2247226-s002.pdf]

1

Supplemental Online Content

Lahitnen P, Juuti A, Luostarinen M, et al. Effectiveness of fecal microbiota transplantation for weight loss in patients with obesity undergoing bariatric surgery: a randomized clinical trial. *JAMA Netw Open*. 2022;5(12):e2247226. doi:10.1001/jamanetworkopen.2022.47226

eFigure 1. Main End Points

eFigure 2. Estimated Marginal Means of %EBMIL by Operation Type

eTable 1. Body Composition

eTable 2. Blood Chemistry

eFigure 3. Blood Chemistry: Variables Associated With Metabolism

eFigure 4. 15D Quality of Life

This supplemental material has been provided by the authors to give readers additional information about their work.

© 2022 Lahtinen P et al. *JAMA Network Open*.

2

eFigure 1. Main End Points

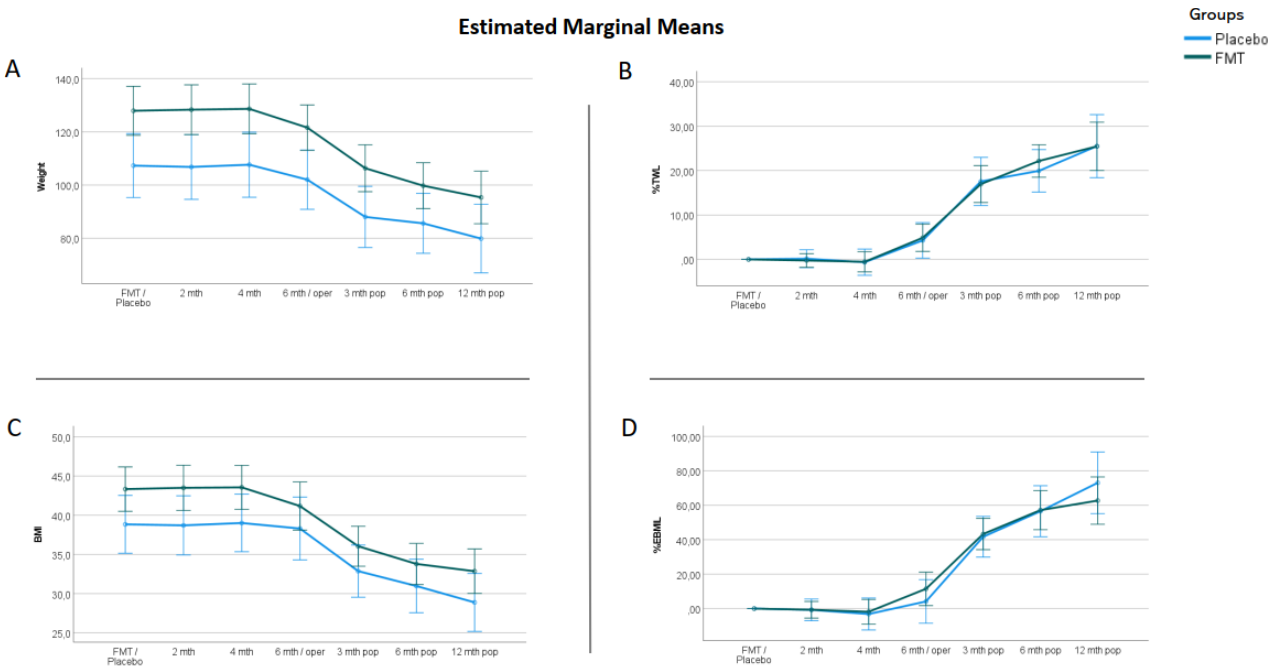

Error bars: 95% CI

A The mean weight of the placebo group (blue) and the FMT group (green) at each measurement point in the study; the difference between the groups was not statistically significant. B The mean total weight loss percentage (%TWL) in the placebo group (blue) and the FMT group (green) at each measurement point in the study; the difference between the groups was not statistically significant. C The mean BMI of the placebo group (blue) and the FMT group (green) at each measurement point in the study; the difference between the groups was not statistically significant. D The mean excess BMI loss percentage (%EBMIL) in the placebo group (blue) and the FMT group (green) at each measurement point in the study; the difference between the groups was not statistically significant. The estimated marginal means were analyzed using repeated measures analysis of variance.

© 2022 Lahtinen P et al. *JAMA Network Open.*

3

eFigure 2. Estimated Marginal Means of %EBMIL by Operation Type

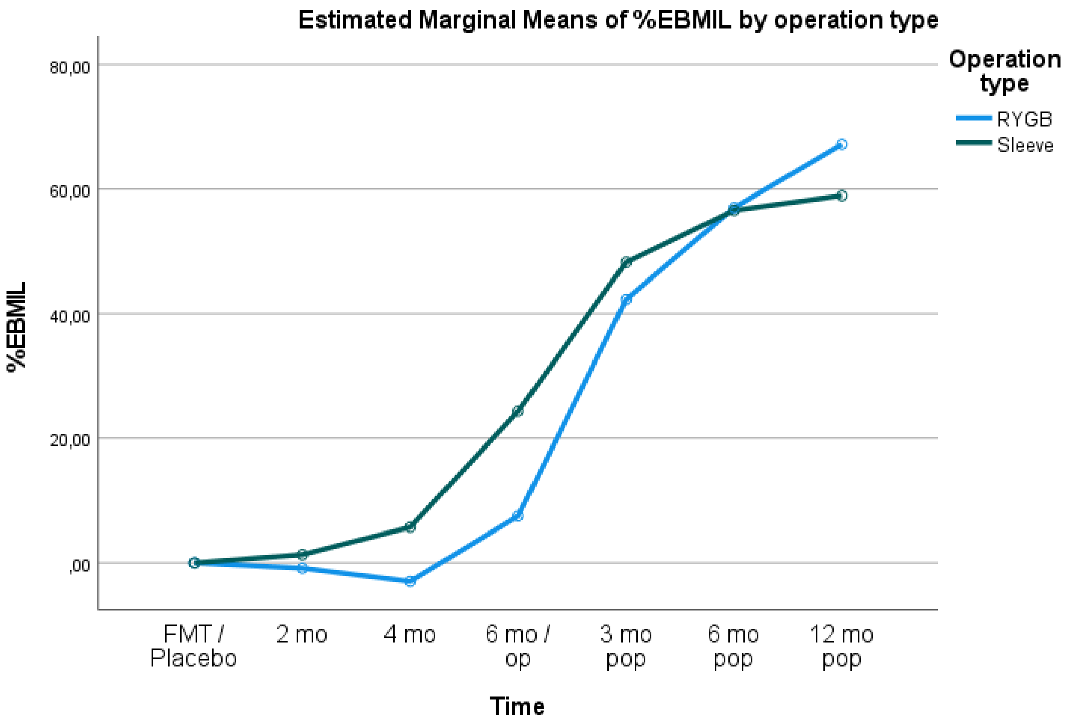

Only two out of the four patients who underwent sleeve operation attended every study visit and were thus included in this analysis. Three out of four patients who underwent sleeve operation attended the final visit and they were all in the FMT group. The final mean %EMBIL of these three patients was 49.7% (95% CI, 68.7-31.1) compared to 67.4% (95% CI, 76.9 - 57.6) in the patients who underwent RYGB operation and attended the final visit (N=31). When analyzed without the sleeve operated patients, the mean %EBMIL at 12 months after the surgery increased from 62.9% (95% CI, 77.6-48.2, P < .001) to 65.4% (95% CI, 79.8-50.6, P < .001) in the FMT group compared to 69.4% (95% CI, 81.5-57.4, P < .001) in the placebo group. The %EBMIL remained statistically insignificant between the groups after the exclusion of sleeve operated patients.

© 2022 Lahtinen P et al. *JAMA Network Open.*

4

eTable 1. Body Composition

	Visceral adiposity (%)					Fat percentage (%)					Muscle mass (kg)				
	FMT		Placebo			FMT		Placebo			FMT		Placebo		
	Mean	SD	Mean	SD	*P*-value	Mean	SD	Mean	SD	*P*-value	Mean	SD	Mean	SD	*P*-value
Baseline	18,4	5,6	16,0	5,7	0,19	43,9	5,5	43,0	5,1	0,60	66,8	10,0	65,4	15,1	0,73
2 mo	18,1	5,6	16,7	5,6	0,43	43,8	5,4	44,4	4,5	0,70	66,4	10,4	64,7	14,1	0,65
4 mo	18,4	5,9	15,9	4,7	0,17	44,1	5,4	45,5	4,8	0,41	66,4	11,0	61,0	14,1	0,20
3 mo pop	13,6	4,7	11,7	3,6	0,19	38,4	7,4	39,3	5,2	0,69	60,6	10,2	57,3	13,9	0,42
6 mo pop	12,5	4,6	10,5	3,2	0,17	35,9	8,4	36,9	4,8	0,68	59,9	10,3	55,7	11,6	0,28
12 mo pop	11,5	4,9	9,3	3,8	0,17	34,3	10,1	34,5	4,8	0,94	59,5	10,9	52,6	11,5	0,09

Abbreviations: FMT, fecal microbiota transplantation; mo, months; pop, post-operation.

© 2022 Lahtinen P et al. *JAMA Network Open*.

5

eTable 2. Blood Chemistry

	FMT	Placebo	*P* value
Hb level at baseline (mg/l)	143.1 (12.9)	140.4 (12.0)	0.49
Hb level at 2 months (mg/l)	143.1 (13.8)	141.2 (11.7)	0.63
Hb level at 4 months (mg/l)	142.4 (13.3)	141.2 (13.6)	0.77
Hb level at 6 months (mg/l)	144.1 (10.3)	142.5 (11.2)	0.65
Hb level at 3 months after surgery (mg/l)	136.7 (10.5)	140.4 (10.2)	0.29
Hb level at 6 months after surgery (mg/l)	136.4 (11.1)	138.5 (11.1)	0.57
Hb at 12 months after surgery (mg/l)	138.0 (13.7)	138.7 (8.6)	0.86
Leukocyte count at baseline (10E9/l)	6.7 (0.9)	7.1 (1.4)	0.20
Leukocyte count at 2 months (10E9/l)	6.4 ((1.3)	7.2 (2.1)	0.18
Leukocyte count at 4 months (10E9/l)	6.2 (1.4)	7.8 (3.3)	**0.05**
Leukocyte count at 6 months (10E9/l)	6.1 (1.6)	6.6 (1.4)	0.39
Leukocyte count at 3 months after surgery (10E9/l)	6.1 (2.7)	6.4 (1.1)	0.69
Leukocyte count at 6 months after surgery (10E9/l)	5.7 (1.4)	6.6 (1.4)	0.69
Leukocyte count at 12 months after surgery (10E9/l)	5.3 (1.3)	5.8 (0.9)	0.13
Thrombocyte count at baseline (10E9/l)	249.7 (37.4)	299.0 (61.9)	**0.04**
Thrombocyte count at 2 months (10E9/l)	274.6 (131.5)	302.2 (63.1)	0.41
Thrombocyte count at 4 months (10E9/l)	244.9 (37.0)	289.9 (55.3)	**0.04**
Thrombocyte count at 6 months (10E9/l)	244.7 (35.8)	272.0 (54.8)	0.80
Thrombocyte count at 3 months after surgery (10E9/l)	237.6 (32.7)	284.4 (59.5)	**0.01**
Thrombocyte count at 6 months after surgery (10E9/l)	237.5 (36.4)	286.1 (24.3)	**0.001**
Thrombocyte count at 12 months after surgery (10E9/l)	244.8 (38.4)	279.3 (54.3)	**0.04**
C-reactive protein level at baseline (mg/l)	5.2 (4.8)	5.8 (3.3)	0.65
C-reactive protein level at 2 months (mg/l)	4.7 (3.2)	6.5 (3.4)	0.09
C-reactive protein level at 4 months (mg/l)	7.2 (6.4)	6.7 (3.8)	0.79
C-reactive protein level at 6 months (mg/l)	5.1 (4.7)	5.4 (3.5)	0.81
C-reactive protein level at 3 months after surgery (mg/l)	14.2 (49.6)	2.8 (1.3)	0.34
C-reactive protein level at 6 months after surgery (mg/l)	2.6 (1.5)	3.2 (2.6)	0.39
C-reactive protein level at 12 months after surgery (mg/l)	2.3 (1.4)	2.6 (1.6)	0.61
ESR at baseline (mm/h)	10.6 (8.1)	14.8 (9.7)	0.15
ESR at 2 months (mm/h)	11.3 (9.3)	16.2 (11.9)	0.09
ESR at 4 months (mm/h)	7.2 (6.4)	6.7 (3.8)	0.79
ESR at 6 months (mm/h)	5.1 (4.7)	5.4 (3.5)	0.87
ESR at 3 months after surgery (mm/h)	14.2 (49.6)	2.8 (1.3)	0.72
ESR at 6 months after surgery (mm/h)	9.3 (7.9)	11.4 (7.6)	0.41
ESR at 12 months after surgery (mm/h)	8.7 (8.4)	7.2 (3.3)	0.52
Potassium level at baseline (mmol/l)	4.0 (0.2)	3.9 (0.2)	0.19
Potassium level at 2 months (mmol/l)	4.0 (0.2)	4.2 (0.7)	0.15
Potassium level at 4 months (mmol/l)	4.0 (0.3)	3.9 (0.3)	0.10

© 2022 Lahtinen P et al. *JAMA Network Open.*

6

Potassium level at 6 months (mmol/l)	3.9 (0.2)	3.8 (0.3)	0.54
Potassium level at 3 months after surgery (mmol/l)	3.8 (0.3)	3.8 (0.3)	1.00
Potassium level at 6 months after surgery (mmol/l)	3.8 (0.2)	3.8 (0.3)	0.44
Potassium level at 12 months after surgery (mmol/l)	3.8 (0.3)	3.7 (0.2)	0.32
Sodium level at baseline (mmol/l)	139.2 (1.9)	139.9 (2.5)	0.32
Sodium level at 2 months (mmol/l)	139.6 (1.7)	140.1 (2.2)	0.39
Sodium level at 4 months (mmol/l)	133.5 (27.4)	139.3 (1.4)	0.38
Sodium level at 6 months (mmol/l)	139.3 (1.7)	139.4 (2.6)	0.92
Sodium level at 3 months after surgery (mmol/l)	140.3 (2.2)	141.1 (2.1)	0.27
Sodium level at 6 months after surgery (mmol/l)	148.8 (1.6)	140.9 (2.0)	0.80
Sodium level at 12 months after surgery (mmol/l)	141.0 (2.9)	140.8 (2.2)	0.83
Creatinine level at baseline (umol/l)	67.0 (12.7)	65.8 (12.7)	0.77
Creatinine level at 2 months (umol/l)	66.1 (8.6)	65.3 (13.4)	0.80
Creatinine level at 4 months (umol/l)	69.3 (13.2)	66.1 (14.0)	0.46
Creatinine level at 6 months (umol/l)	69.9 (14.7)	68.8 (17.7)	0.84
Creatinine level at 3 months after surgery (umol/l)	65.7 (13.0)	63.6 (8.8)	0.57
Creatinine level at 6 months after surgery (umol/l)	66.4 (12.7)	64.2 (11.3)	0.59
Creatinine level at 12 months after surgery (umol/l)	63.9 (11.6)	63.2 (12.9)	0.86
Albumin level at baseline (g/l)	36.5 (2.7)	37.4 (3.2)	0.37
Albumin level at 2 months (g/l)	36.8 (3.0)	36.9 (3.0)	0.88
Albumin level at 4 months (g/l)	36.8 (3.0)	36.9 (3.0)	0.15
Albumin level at 6 months (g/l)	37.7 (2.2)	39.1 (2.6)	0.11
Albumin level at 3 months after surgery (g/l)	36.3 (3.5)	37.7 (3.0)	0.57
Albumin level at 6 months after surgery (g/l)	36.4 (3.2)	37.0 (2.9)	0.54
Albumin level at 12 months after surgery (g/l)	38.2 (2.8)	38.1 (3.0)	0.94
Fasting glucose level at baseline (mmol/l)	6.8 (2.5)	6.1 (1.1)	0.25
Fasting glucose level at 2 months (mmol/l)	6.9 (3.1)	6.2 (1.2)	0.36
Fasting glucose level at 4 months (mmol/l)	6.9 (3.0)	8.4 (9.7)	0.51
Fasting glucose level at 6 months (mmol/l)	6.3 (1.4)	5.9 (1.1)	0.29
Fasting glucose level at 3 months after surgery (mmol/l)	5.8 (1.3)	5.7 (0.8)	0.71
Fasting glucose level at 6 months after surgery (mmol/l)	6.3 (1.4)	5.9 (1.1)	0.65
Fasting glucose level at 12 months after surgery (mmol/l)	5.6 (0.9)	5.5 (0.6)	0.71
HbA1c level at baseline (mmol/mol)	42.3 (13.6)	40.3 (9.7)	0.61
HbA1c level at 2 months (mmol/mol)	42.2 (15.6)	40.3 (8.2)	0.64
HbA1c level at 4 months (mmol/mol)	42.8 (17.5)	40.4 (9.8)	0.61
HbA1c level at 6 months (mmol/mol)	39.7 (11.3)	38.8 (8.3)	0.78
HbA1c level at 3 months after surgery (mmol/mol)	35.8 (7.1)	36.5 (6.0)	0.76
HbA1c level at 6 months after surgery (mmol/mol)	36.6 (6.1)	36.4 (4.7)	0.91
HbA1c level at 12 months after surgery (mmol/mol)	35.2 (4.8)	35.5 (5.2)	0.88
INR at baseline	1.0 (0.1)	1.0 (0.1)	0.97
INR at 2 months	1.0 (0.1)	1.0 (0.1)	0.93
INR at 4 months	1.0 (0.1)	1.0 (0.1)	0.50
INR at 6 months	1.1 (0.1)	1.0 (0.1)	0.25
INR at 3 months after surgery	1.1 (0.1)	1.1 (0.1)	0.41
INR at 6 months after surgery	1.1 (0.1)	1.1 (0.1)	0.94

© 2022 Lahtinen P et al. *JAMA Network Open.*

7

INR at 12 months after surgery	1.1 (0.1)	1.0 (0.1)	0.34
TfR level at baseline (mg/l)	3.3. (0.8)	3.1 (0.6)	0.57
TfR level at 2 months (mg/l)	3.3 (0.8)	3.4 (1.1)	0.82
TfR level at 4 months (mg/l)	3.3 (0.9)	3.1 (0.7)	0.44
TfR level at 6 months (mg/l)	3.3 (0.8)	3.0 (0.5)	0.26
TfR level at 3 months after surgery (mg/l)	3.3 (0.8)	2.9 (0.5)	**0.05**
TfR level at 6 months after surgery (mg/l)	3.2 (0.8)	2.8 (0.6)	0.11
TfR level at 12 months after surgery (mg/l)	3.3 (0.9)	2.7 (0.8)	0.09
ALP level at baseline (U/l)	70.0 (15.0)	75.1 (18.6)	0.34
ALP level at 2 months (U/l)	70.4 (15.9)	77.1 (19.8)	0.25
ALP level at 4 months (U/l)	74.4 (23.2)	74.7 (20.3)	0.97
ALP level at 6 months (U/l)	62.2 (12.5)	67.2 (14.3)	0.27
ALP level at 3 months after surgery (U/l)	74.4 (20.8)	80.7 (18.5)	0.34
ALP level at 6 months after surgery (U/l)	79.2 (22.7)	86.7 (23.1)	0.33
ALP level at 12 months after surgery (U/l)	76.3 (21.2)	82.5 (24.8)	0.45
ALT level at baseline (U/l)	27.7 (11.6)	42.9 (30.2)	**0.04**
ALT level at 2 months (U/l)	30.5 (15.5)	36.4 (26.9)	0.40
ALT level at 4 months (U/l)	36.2 (33.0)	55.8 (55.2)	0.18
ALT level at 6 months (U/l)	34.1 (13.5)	44.8 (21.8)	0.09
ALT level at 3 months after surgery (U/l)	29.7 (16.7)	32.8 (18.7)	0.61
ALT level at 6 months after surgery (U/l)	33.1 (14.1)	29.7 (12.4)	0.45
ALT level at 12 months after surgery (U/l)	32.9 (20.5)	34.6 (17.1)	0.80
AST level at baseline (U/l)	27.9 (9.9)	29.9 (14.1)	0.60
AST level at 2 months (U/l)	29.1 (13.6)	27.4 (9.3)	0.65
AST level at 4 months (U/l)	29.6 (14.9)	33.8 (16.8)	0.41
AST level at 6 months (U/l)	27.9 (7.4)	29.6 (10.3)	0.58
AST level at 3 months after surgery (U/l)	28.1 (11.3)	24.7 (7.3)	0.28
AST level at 6 months after surgery (U/l)	28.5 (7.7)	24.8 (5.5)	0.11
AST level at 12 months after surgery (U/l)	28.3 (11.7)	29.5 (17.6)	0.83
Urate level at baseline (µmol/l)	349.5 (70.6)	308.3 (94.7)	0.12
Urate level at 2 months (µmol/l)	342.6 (55.5)	338.6 (77.9)	0.85
Urate level at 4 months (µmol/l)	363.5 (67.4)	331.5 (60.2)	0.14
Urate level at 6 months (µmol/l)	362.5 (59.7)	355.7 (76.4)	0.77
Urate level at 3 months after surgery (µmol/l)	312.9 (50.2)	300.2 (58.2)	0.48
Urate level at 6 months after surgery (µmol/l)	306.1 (66.8)	297.0 (78.0)	0.71
Urate level at 12 months after surgery (µmol/l)	295.4 (55.9)	293.7 (73.2)	0.94
Bilirubin level at baseline (µmol/l)	10.0 (4.2)	9.5 (4.3)	0.70
Bilirubin level at 2 months (µmol/l)	9.9 (5.1)	10.2 (4.9)	0.85
Bilirubin level at 4 months (µmol/l)	10.0 (3.9)	9.8 3.7)	0.89
Bilirubin level at 6 months (µmol/l)	11.5 (3.2)	10.2 (4.8)	0.35
Bilirubin level at 3 months after surgery (µmol/l)	13.1 (5.9)	12.6 (5.2)	0.79
Bilirubin level at 6 months after surgery (µmol/l)	12.3 (5.6)	11.7 (5.3)	0.74
Bilirubin level at 12 months after surgery (µmol/l)	12.2 (5.6)	11.0 (3.9)	0.50
Cholesterol level at baseline (mmol/l)	4.6 (0.7)	4.9 (0.7)	0.17
Cholesterol level at 2 months (mmol/l)	4.7 (0.7)	5.1 (0.6)	0.17

© 2022 Lahtinen P et al. *JAMA Network Open.*

8

Cholesterol level at 4 months (mmol/l)	4.5 (0.7)	5.1 (0.8)	**0.02**
Cholesterol level at 6 months (mmol/l)	4.2 (0.7)	4.3 (0.6)	0.59
Cholesterol level at 3 months after surgery (mmol/l)	4.0 (0.6)	4.2 (0.6)	0.34
Cholesterol level at 6 months after surgery (mmol/l)	4.2 (0.7)	4.2 (0.5)	1.00
Cholesterol level at 12 months after surgery (mmol/l)	4.4 (0.7)	4.4 (0.6)	0.97
HDL level at baseline (mmol/l)	1.2 (0.3)	1.2 (0.3)	0.96
HDL level at 2 months (mmol/l)	1.2 (0.2)	1.2 (0.3)	0.55
HDL level at 4 months (mmol/l)	1.2 (0.2)	1.3 (0.3)	0.11
HDL level at 6 months (mmol/l)	1.1 (0.2)	1.2 (0.3)	0.51
HDL level at 3 months after surgery (mmol/l)	1.2 (0.2)	1.2 (0.3)	0.61
HDL level at 6 months after surgery (mmol/l)	1.3 (0.3)	1.3 (0.3)	0.66
HDL level at 12 months after surgery (mmol/l)	1.5 (0.2)	1.5 (0.3)	0.73
LDL level at baseline (mmol/l)	3.0 (0.7)	3.3 (0.7)	0.17
LDL level at 2 months (mmol/l)	3.1 (0.7)	3.4 (0.6)	0.23
LDL level at 4 months (mmol/l)	3.0 (0.6)	3.3 (0.8)	0.19
LDL level at 6 months (mmol/l)	2.8 (0.6)	2.8 (0.6)	0.83
LDL level at 3 months after surgery (mmol/l)	2.5 (0.6)	2.6 (0.6)	0.62
LDL level at 6 months after surgery (mmol/l)	2.7 (0.7)	2.6 (0.6)	0.69
LDL level at 12 months after surgery (mmol/l)	2.7 (0.7)	2.6 (0.6)	0.75
Triglyceride level at baseline (mmol/l)	2.0 (1.4)	1.7 (1.1)	0.94
Triglyceride level at 2 months (mmol/l)	2.0 (1.4)	1.7 (1.1)	0.43
Triglyceride level at 4 months (mmol/l)	1.6 (1.2)	1.9 (1.6)	0.54
Triglyceride level at 6 months (mmol/l)	1.2 (0.6)	1.3 (0.6)	0.70
Triglyceride level at 3 months after surgery (mmol/l)	1.2 (0.4)	1.3 (0.8)	0.32
Triglyceride level at 6 months after surgery (mmol/l)	1.1 (0.4)	1.2 (0.5)	0.32
Triglyceride level at 12 months after surgery (mmol/l)	1.0 (0.4)	1.1 (0.5)	0.89

© 2022 Lahtinen P et al. *JAMA Network Open.*

9

eFigure 3. Blood Chemistry: Variables Associated With Metabolism

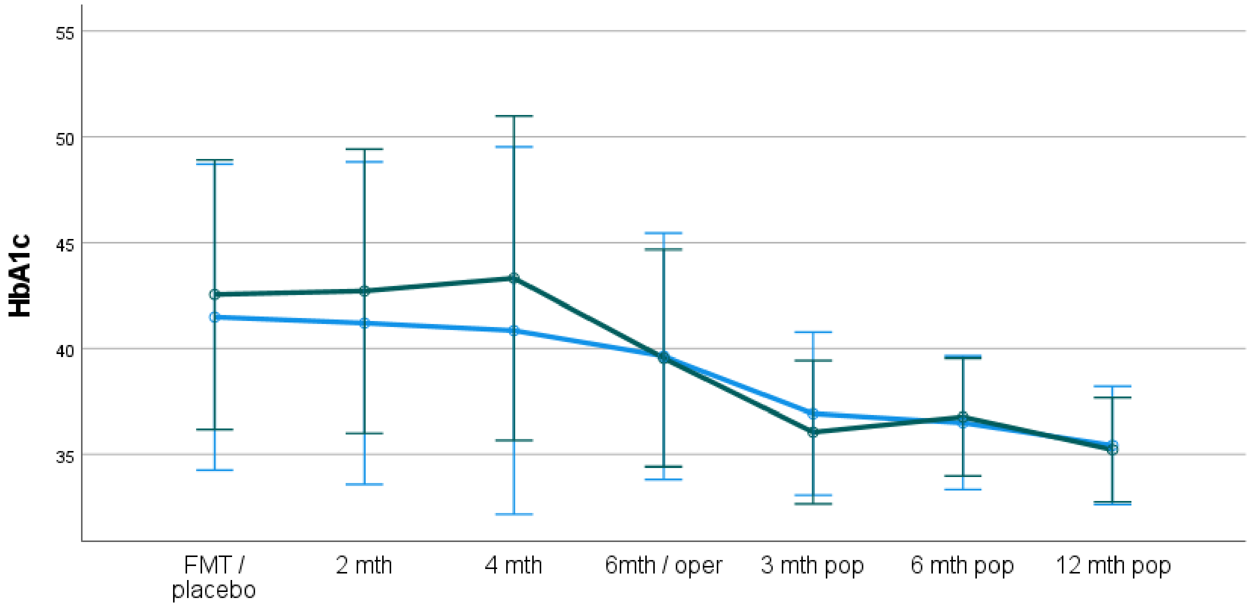

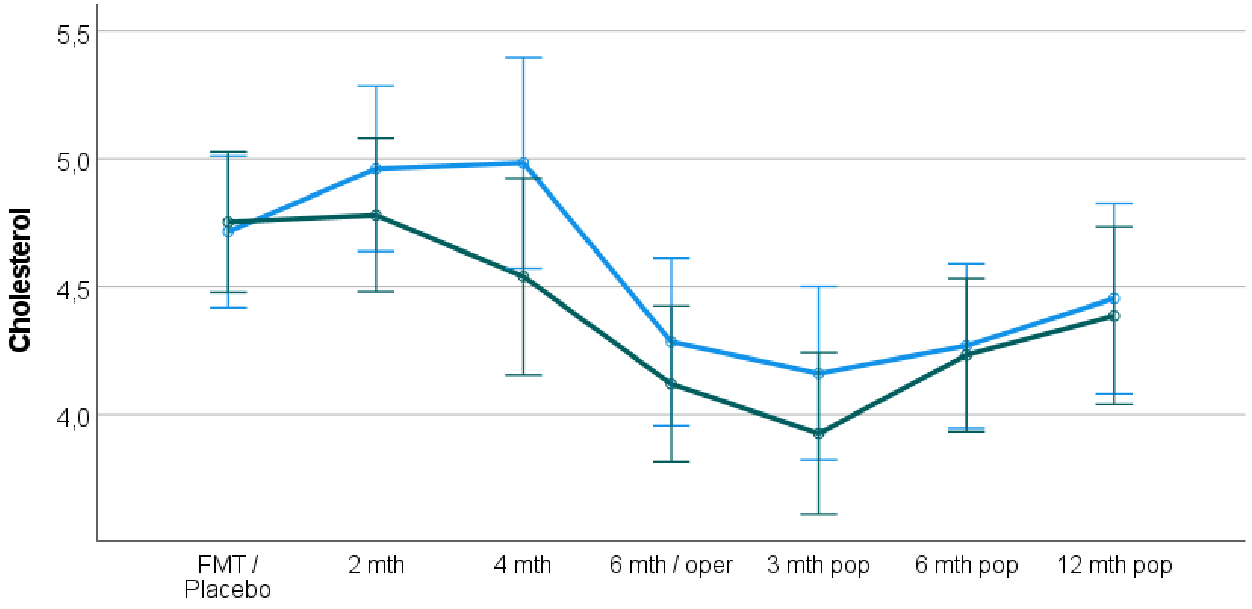

© 2022 Lahtinen P et al. *JAMA Network Open.*

10

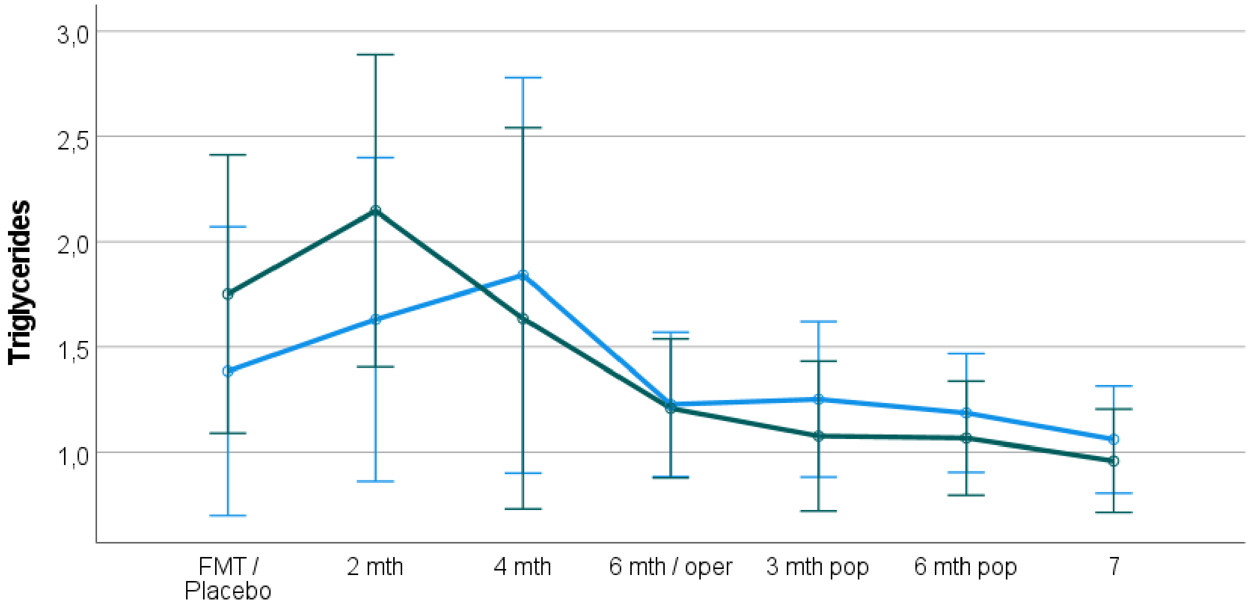

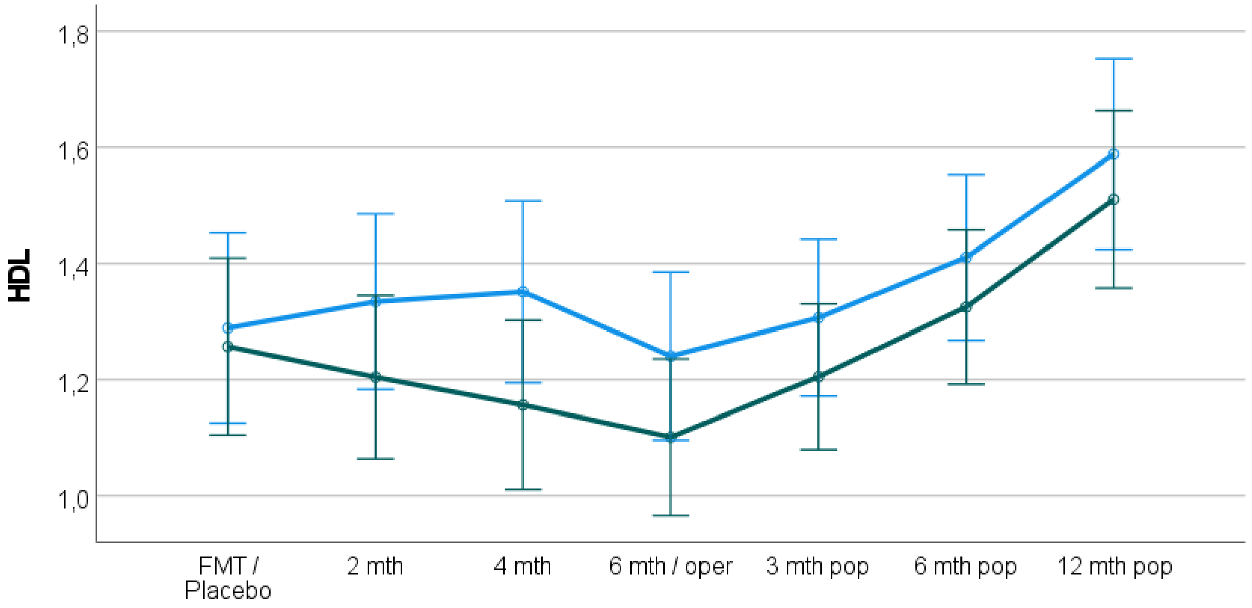

© 2022 Lahtinen P et al. *JAMA Network Open.*

11

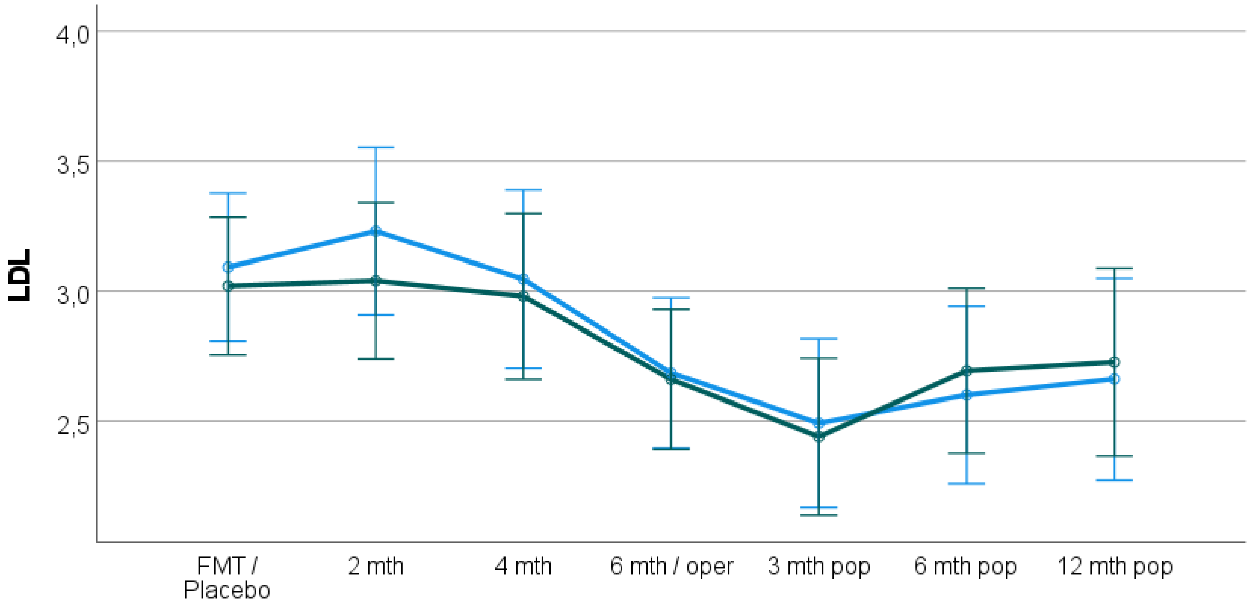

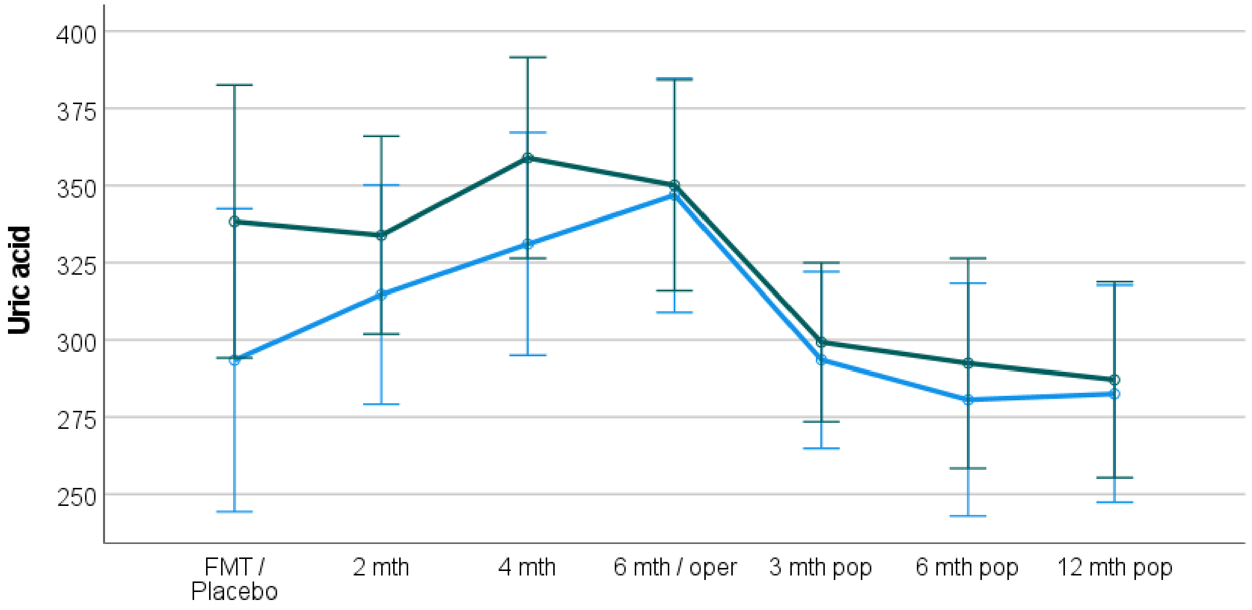

The figure series presents the estimated marginal means of HbA1c, hemoglobin-A1c; Cholesterol; HDL, high density lipoprotein; LDL, low density lipoprotein and uric acid. Green line, FMT group; blue line, placebo group.

© 2022 Lahtinen P et al. *JAMA Network Open*.

12

eFigure 4. 15D Quality of Life

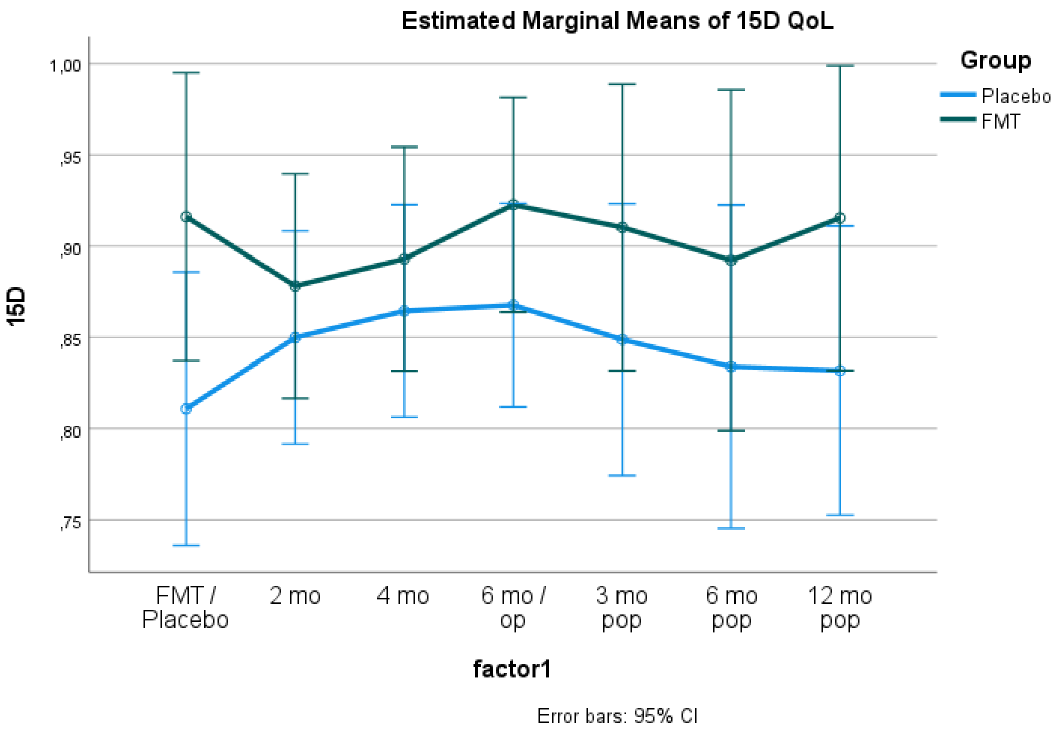

Abbreviations: 15D, 15 dimensions; QoL, quality of life; FMT, fecal microbiota transplantation; mo, months; pop, post-operation.

© 2022 Lahtinen P et al. *JAMA Network Open*.
