# Supplementary material for: Effectiveness of Fecal Microbiota Transplantation for Weight Loss in Patients With Obesity Undergoing Bariatric Surgery: A Randomized Clinical Trial
Source: JAMA Netw Open. 2022 Dec 16;5(12):e2247226. doi: 10.1001/jamanetworkopen.2022.47226 (PMC9856235; doi:10.1001/jamanetworkopen.2022.47226)
Supplement: Supplement 3. — Data Sharing Statement [file jamanetwopen-e2247226-s003.pdf]

Data Sharing Statement

Lahtinen. Effectiveness of Fecal Microbiota Transplantation for Weight Loss in Patients With Obesity Undergoing Bariatric Surgery. *JAMA Netw Open*. Published December 16, 2022. doi:10.1001/jamanetworkopen.2022.47226

Data
Data available: Yes
Data types: Deidentified participant data
How to access data: perttu.lahtinen@phhyky.fi
When available: With publication

Supporting Documents
Document types: None

Additional Information
Who can access the data: researchers whose proposed use of the data has been approved
Types of analyses: for any purpose
Mechanisms of data availability: after approval of a proposal
